# Metformin treatment for 8 days impacts multiple intestinal parameters in high-fat high-sucrose fed mice

**DOI:** 10.1038/s41598-021-95117-0

**Published:** 2021-08-17

**Authors:** Amélie Bravard, Céline Gérard, Clémence Defois, Bérengère Benoit, Kassem Makki, Emmanuelle Meugnier, Dominique Rainteau, Jennifer Rieusset, Murielle Godet, Hubert Vidal

**Affiliations:** 1 Laboratoire CarMeN, INSERM U1060, INRAE U1397, Université de Lyon, Université Claude Bernard Lyon1, 69600 Oullins, France; 2 Centre de Recherche Saint-Antoine, INSERM U938, Sorbonne Université, 75012 Paris, France; 3 Present Address: Department of Molecular and Clinical Medicine/Wallenberg Laboratory, Institute of Medicine, University of Gothenburg and Sahlgrenska University Hospital, Gothenburg, Sweden

**Keywords:** Metabolism, Metabolic diseases, Diabetes, Type 2 diabetes, Physiology, Endocrinology, Endocrine system and metabolic diseases, Diabetes

## Abstract

Although the mechanism of action of the antidiabetic drug metformin is still a matter of discussions, it is well accepted that the gut plays an important role. To gain more insights into the mechanisms occurring in the different regions of the intestine, adult male mice were fed a high-fat-high sucrose (HFS) diet for 8 days and treated with metformin by gavage (300 mg/day/kg body weight) during the HFS diet. Metformin counteracted HFS diet-induced overexpression of a network of genes involved in the transport of glucose and fatty acids in the different regions of the small intestine. It also induced beneficial modification of secondary bile acid profile in the caecum, with a reduction of deoxycholic acid and lithocholic acid levels and increased abundance of ursodeoxycholic acid and tauroursodeoxycholic acid, potentially leading to FRX inhibition. In parallel, metformin treatment was associated with specific changes of the microbiota composition in the lumen of the different regions of the intestine. Metformin induced a marked increase in the abundance of *Akkermansia muciniphila* in the lumen all along the gut and counteracted the effects of HFS diet on the abundances of some bacterial groups generally associated with metabolic disturbances (f-Lachnospiraceae, f-Petostreptococcaceae, g-*Clostidium*). Therefore, the present work clearly emphasises the role of all the regions of the intestinal tract in the beneficial action of the antidiabetic drug metformin in a prediabetic mouse model.

## Introduction

The oral antidiabetic drug metformin (1,1-dimethylbiguanide hydrochloride) is the first line therapy of type 2 diabetes mellitus (T2DM). Despite its use for more than 60 years, the mechanisms contributing to its effects on blood glucose levels are still subject of debates^1,2^. Clinical studies have demonstrated that metformin acts primarily by the reduction of exaggerated endogenous glucose production in diabetic patients, especially through a decrease of hepatic gluconeogenesis rate^3^. Animal studies and in vitro experiments in hepatocytes revealed an inhibition of complex I of the mitochondrial electron transfer chain and activation of AMP-activated protein kinase (AMPK), leading to the repression of gluconeogenic gene expression^1,2^. In parallel, several works have suggested that metformin may act primarily by targeting intestinal tract as it can accumulate and reach high concentrations in the small intestine^4^. Furthermore, oral administration of metformin appeared to reduce blood glucose more efficiently than the intravenous route^5^, and a gut-restricted formulation was shown to efficiently decrease glucose levels in T2DM patients^6^. It was also shown that metformin is able to inhibit intestinal glucose absorption in the proximal small intestine^7^, and to increase glucose uptake and utilization by the enterocytes^8^. Additional modifications of intestinal functions have been reported, including increased production of the incretin hormone glucagon-like peptide 1 (GLP1)^9^, stimulation of Goblet cells specialized in mucin production^10^ and bile acid pool modifications^11^. These effects can be related to a direct action of the drug on intestinal cells or to consequences of a modification of the gut microbiota. Indeed, it is currently well accepted that metformin is able to alter the overall structure and the functions of the gut microbiota^12–15^ and it was shown that metformin can stimulate directly the growth of specific bacterial species, notably *Akkermansia muciniphila*^10,13,14^. However, the direct link between the modulation of microbiota and the metabolic actions of metformin is still debated^16^.

To gain more insights into the mechanisms that occur in the different regions of the intestinal tract in response to metformin treatment, we investigated in the present study the changes in a number of biological parameters in different regions of the intestinal tract after a short-term metformin treatment in high-fat high-sucrose (HFS) fed mice. In addition to its effects on the expression of key intestinal genes in different segments (duodenum, jejunum, ileum, colon), we evaluated the impact of the drug on the luminal microbiota composition of each gut segment, and we performed a bile acid profiling in the caecum of the animals. Mice were fed HFS diet for 8 days and were treated with metformin by daily gavage. We found multi-level effects of metformin, leading in one week to a restoration of most of the perturbations induced by HFS diet on gene expression, associated with modifications of the bile acid profile and of the luminal microbiota composition all along the gut.

## Results

### Short metformin treatment prevented metabolic disturbances in HFS fed mice

**Figure 1:**
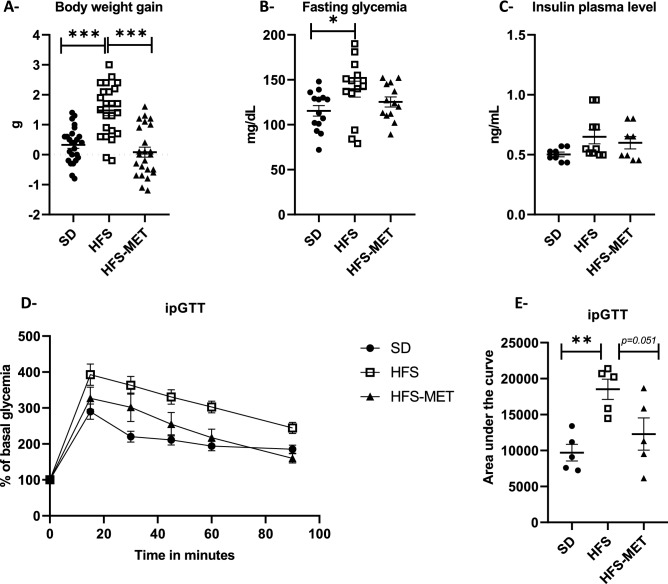
Metformin improves metabolic parameters and glucose tolerance in high fat high sucrose fed mice. (**A**) change in body weight after 8 days of standard diet (SD), high fat high sucrose diet without (HFS) or with metformin supplementation (HFS-MET). (**B,C**) Fasting plasma glucose and insulin concentrations, respectively. (**D**) Glycemic curves during intraperitoneal glucose tolerance test (ipGTT) performed on 5 animals per group, and (**E**) corresponding area under the curves (AUC) of the ipGTT. *p < 0.05, **p < 0.01, ***p < 0.001, determined by ANOVA followed by Tukey’s multiple comparison test.

HFS feeding for a period of 8 days induced metabolic disturbances in adult C57BL/6J male mice, as evidenced by a significant rise in fasting glucose levels and a deterioration of glucose tolerance during ipGTT (Fig. 1). There was also an increase in body weight upon HFS feeding (mean gain of 1.7 ± 0.2 g in HFS vs 0.2 ± 0.1 in SD group). The administration of metformin was able to counteract these alterations, as reflected by the maintenance of normal body weight gain (mean gain of 0.1 ± 0.1 g) and the preservation of glucose tolerance, although the effect of metformin on fasting glucose levels did not reach statistical significance (Fig. 1).

### Short metformin treatment restored the expression of important genes in intestinal regions

**Figure 2:**
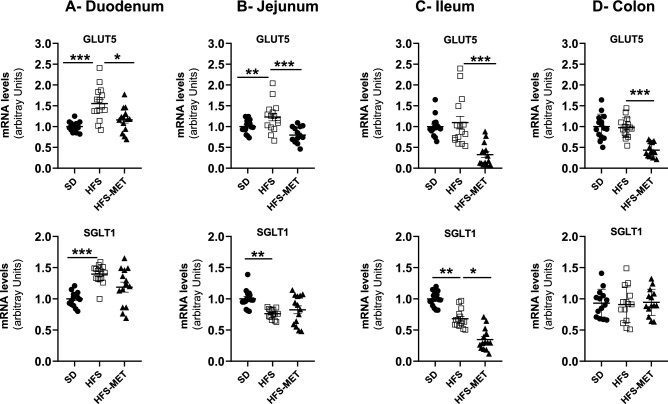
Effects of HFS diet and metformin treatment on apical glucose transporter gene expression in the different regions of the intestine. GLUT5 and SGLT1 mRNA levels were determined by RT-qPCR in (**A**) the duodenum, (**B**) jejunum, (**C**) ileum and (**D**) colon of 15 to 18 mice per group (*SD* standard diet, *HFS* high fat high sucrose diet, *HFS-MET* HFS diet with metformin supplementation). *p < 0.05, **p < 0.01, ***p < 0.001, determined by ANOVA followed by Tukey’s multiple comparison test.

The different intestinal segments express specific patterns of genes allowing a spatial organization of a number of functions along the intestinal tract, such as nutrient digestion and absorption, chylomicron production, bile acid uptake, gut hormone secretion, or immune response and microbe defense. Therefore, we analyzed for each intestinal segment the expression of a specific set of genes after 8 days of HFS feeding with or without metformin. Regarding the genes coding for the apical sugar transporters (Fig. 2), namely the sodium-glucose cotransporter SGLT1(encoded by *Slc5a1*) and the fructose transporter GLUT5 (*Slc2a5*), we found that HFS diet increased GLUT5 and SGLT1 expression in the duodenum, the main region of sugar absorption, while metformin counteracted these effects and significantly reduced GLUT5 expression in all segments of the intestine. We also observed that metformin was able to reduce Glut2 expression in the duodenum (data not shown).

**Figure 3:**
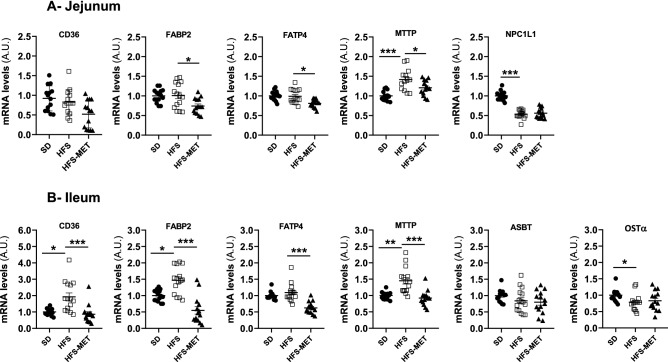
Effects of HFS diet and metformin treatment on the expression of lipid absorption-related genes in the jejunum and the ileum. The mRNA levels of the targeted genes were determined by RT-qPCR in (**A**) the jejunum and (**B**) the ileum of 15 to 18 mice per group (*SD* standard diet, *HFS* high fat high sucrose diet, *HFS-MET* HFS diet with metformin supplementation). *p < 0.05, **p < 0.01, ***p < 0.001, determined by ANOVA followed by Tukey’s multiple comparison test.

Jejunum and ileum are the favored sites for lipid absorption. As shown in Fig. 3, HFS diet feeding significantly increased the gene expression of the main actors of apical fatty acid uptake (FAT/CD36 and FATP4), intracellular fatty acid transport (FA2PB2) and chylomicron synthesis (MTTP) in these two regions, while metformin restored their expression to basal levels (Fig. 3). In contrast, the ileal expressions of the apical sodium-dependent bile acid transporter (ASBT, coded by *Slc10a2*) and of the organic solute transporter-alpha (OSTα, coded by *SLC51B*, which exports bile acid across the enterocyte basolateral membrane), were not significantly affected by HFS diet or by metformin (Fig. 3). The mRNA level of the cholesterol transporter NPC1L1 was strongly decreased upon HFS diet in the jejunum and not modified by metformin treatment (Fig. 3).

**Figure 4:**
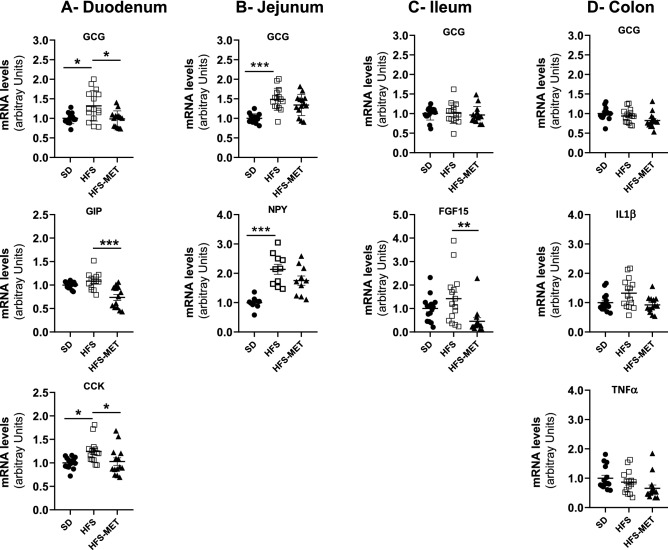
Effects of HFS diet and metformin treatment on the expression of intestinal hormones and peptides in the different regions of the intestine. The mRNA levels of the targeted genes were determined by RT-qPCR in (**A**) the duodenum, (**B**) jejunum, (**C**) ileum and (**D**) colon of 15 to 18 mice per group (*SD* standard diet, *HFS* high fat high sucrose diet, *HFS-MET* HFS diet with metformin supplementation). *p < 0.05, **p < 0.01, ***p < 0.001, determined by ANOVA followed by Tukey’s multiple comparison test.

Results regarding gut hormones and peptides are shown in Fig. 4. We analyzed the expression of the glucagon gene (*Gcg*), encoding GLP-1, in different regions of the gut. HFS diet was associated with an increase in *Gcg* expression in duodenum and jejunum and metformin treatment tended to restore its expression to basal level in duodenum and to slightly decrease it in the colon (Fig. 4). In the other regions, we did not find significant effect of metformin on *Gcg* gene expression. In contrast, the mRNA levels of gastric inhibitory polypeptide (GIP) and cholecystokinin (CCK) in duodenum, of neuropeptide Y (NPY) in jejunum, and of fibroblast growth factor 15 (FGF15) in ileum, which were all increased upon HFS feeding, were restored or down-regulated in the presence of metformin (Fig. 4). Finally, the expression of markers of inflammation (IL1β and TNFα) in the colon was not significantly affected after 8 days of HFS diet or metformin treatment (Fig. 4).

### Metformin modified the bile acid pool

**Figure 5:**
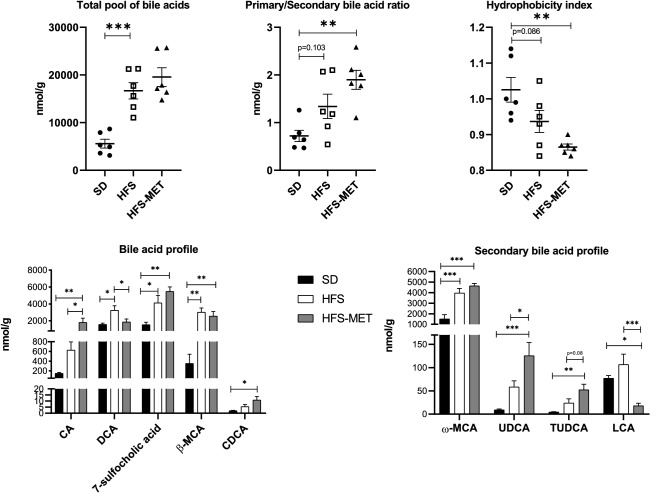
Metformin treatment impacts bile acid pool in the ceacum of the mice. Bile acid molecular species concentrations were measured by HPLC coupled to tandem mass spectrometry (HPLC–MS/MS) in caecum of 6 mice per group (*SD* standard diet, *HFS* high fat high sucrose diet, *HFS-MET* HFS diet with metformin supplementation). *p < 0.05, **p < 0.01, ***p < 0.001, determined by ANOVA followed by Tukey’s multiple comparison test.

Bile acid profiling was performed on the caecum at the end of the treatment periods. As shown in Fig. 5, 8 days of HFS diet significantly increased total bile acid levels. Even though metformin did not modify total bile acid levels compared the HFS group, the treatment tended to increase primary over secondary bile acid ratio and to decrease the hydrophobicity index (Fig. 5).

More specifically, bile acid analysis revealed an increase in cholic acid (CA) levels and a reduction in its main secondary metabolite deoxycholic acid (DCA) upon metformin treatment, whereas the amount of 7-sulfocholic acid was not affected (HFS-MET vs HFS) (Fig. 5). The murine bile acids, beta-muricholic acid (β-MCA) and omega-muricholic acid (ω-MCA) as well as chenodeoxycholic acid (CDCA) were increased by HFS diet, but were not significantly affected by metformin compared to HFS (Fig. 5). Interestingly, the secondary metabolites of CDCA were significantly affected by metformin. We observed a significant increase in ursodeoxycholic acid (UDCA) and tauroursodeoxycholic acid (TUDCA) while lithocholic acid (LCA) levels were decreased by the treatment (Fig. 5). Of note, the relative abundance of conjugated bile acids (tauro-, or sulfo-conjugated) was not significantly affected by metformin (data not shown).

### Metformin modified the luminal microbiota composition in all the regions of the intestine

**Figure 6:**
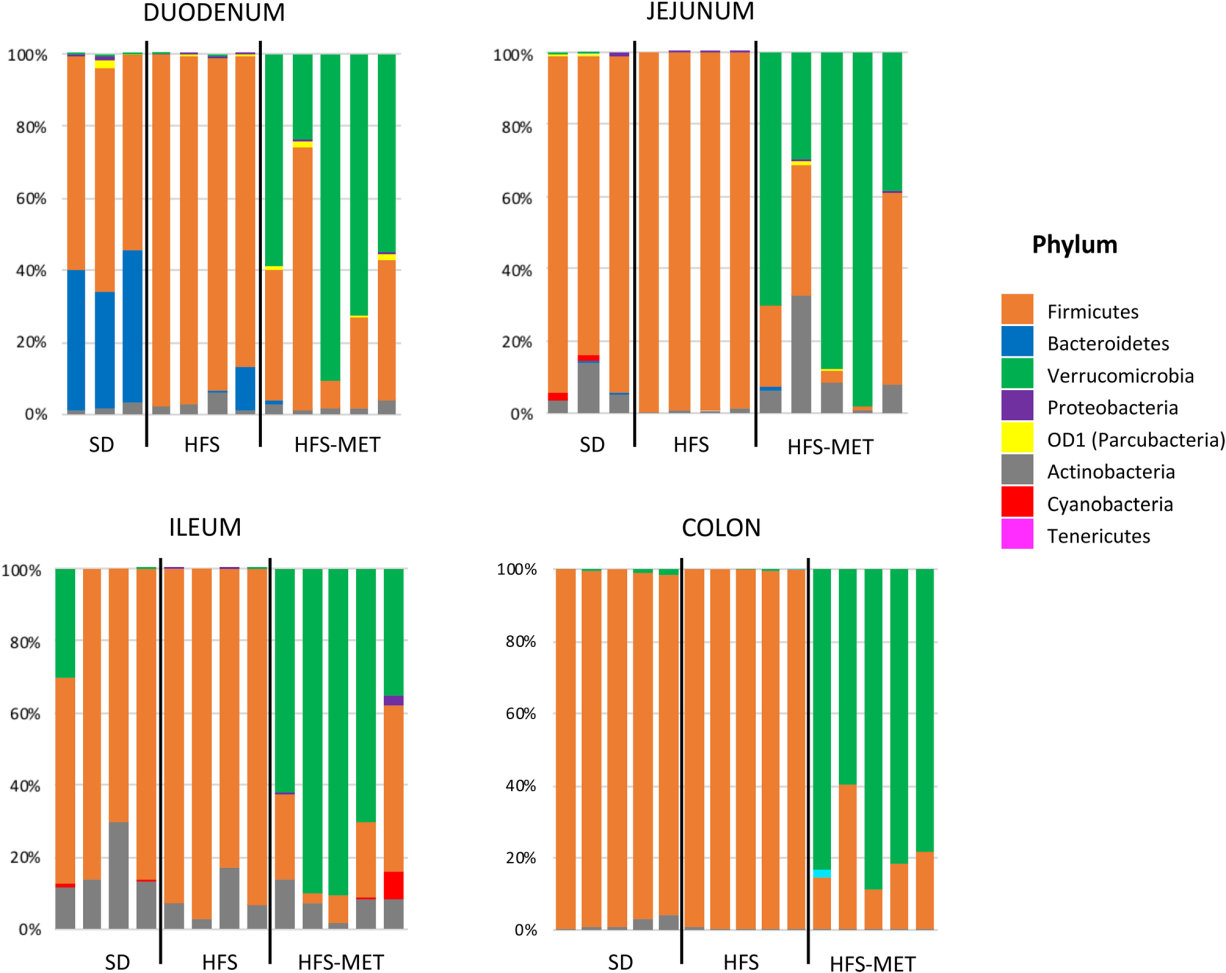
Metformin modifies luminal microbiota composition in the different region of the intestine. Microbiota composition was determined by 16S rRNA sequencing in luminal samples form the duodenum, jejunum, ileum and colon of animals of the 3 groups (*SD* standard diet, *HFS* high fat high sucrose diet, *HFS-MET* HFS diet with metformin supplementation). The figure represents the relative abundance of the main bacterial phyla measured in the luminal samples of individual mice.

**Figure 7:**
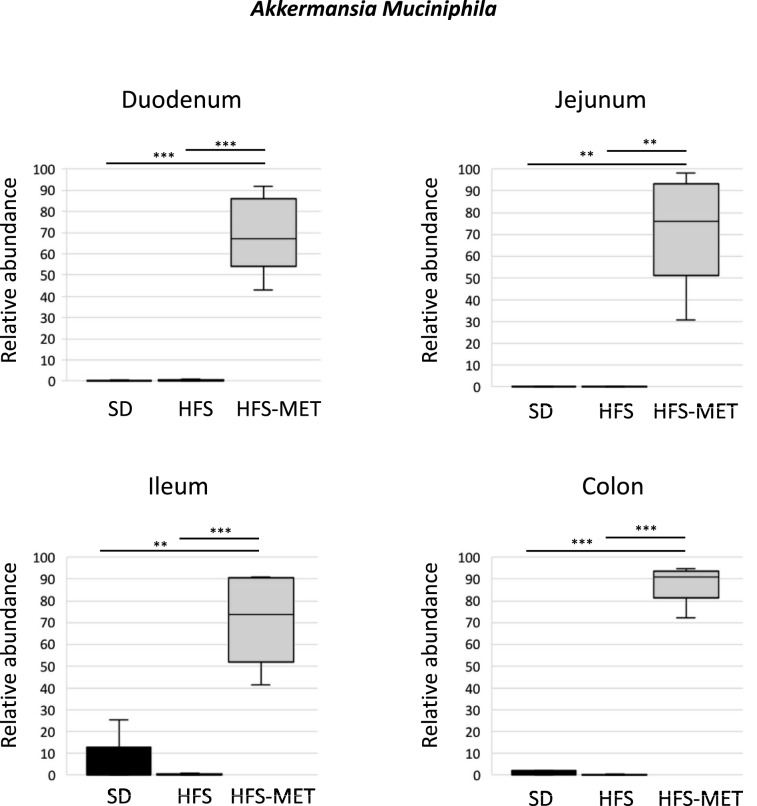
Metformin increases *Akkermansia Muciniphila* abundance in the intestinal lumen all along the gut. Microbiota composition was determined by 16S rRNA sequencing in luminal samples form the duodenum, the jejunum, the ileum and the colon in the different groups (*SD* standard diet, *HFS* high fat high sucrose diet, *HFS-MET* HFS diet with metformin supplementation). The figure represents the relative abundance of *Akkermansia Muciniphila* species. **p < 0.01, ***p < 0.001, determined by ANOVA followed by Tukey’s multiple comparison test.

**Figure 8:**
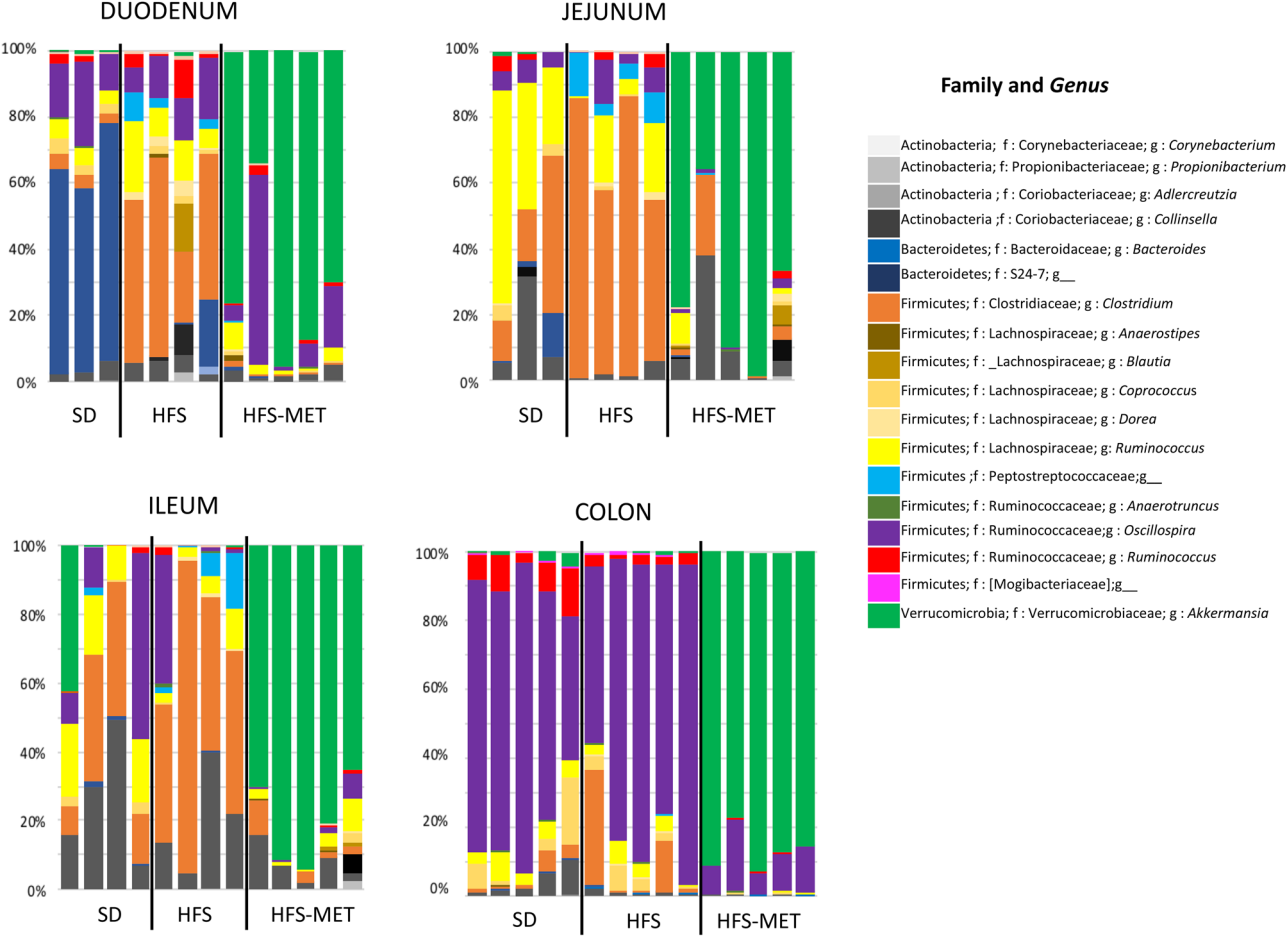
Metformin induces changes of the luminal microbiota composition in the different region of the intestine. Microbiota composition was determined by 16S rRNA sequencing in luminal samples form the duodenum, jejunum, ileum and colon of animals of the 3 groups (*SD* standard diet, *HFS* high fat high sucrose diet, *HFS-MET* HFS diet with metformin supplementation). The figure represents the relative abundance of the main measurable bacterial families and genera in the luminal samples of individual mice.

The number of operational taxonomic units (OTUs) measured in the luminal effluents in the duodenum, the jejunum and the ileum, was similar between groups, with a trend for a lower number of OTUs in the HFS diet group (p = 0.049 vs SD group), not restored by metformin treatment (Sup. Fig. S1). Despite the fact that the bacterial richness appeared globally not modified after 8 days of HFS diet or metformin treatment, we observed a significant impact of metformin at the phylum level in the different regions of the intestinal tract (Fig. 6). While animals fed the HFS diet displayed a slight increase in the proportion of Firmicutes in all segments, especially the duodenum, metformin treatment induced a spectacular increase in the abundance of Verrucomicrobia all along the intestinal tract (Fig. 6). Analysis performed at the genus level revealed that 60% of the sequenced bacteria belonging to this phylum corresponded to *Akkermansia muciniphila,* as shown in Fig. 7. Furthermore, additional analyses indicated that HFS diet and metformin treatment were associated with important modifications at the family and genus levels (Fig. 8, Sup. Fig. S2). We observed increased abundance of *Clostridium* (belonging to the Clostridiaceae family) in all the region of small intestine upon HFS feeding, which was significantly prevented by metformin (Sup. Fig. S2). This effect of metformin treatment on *Clostridium* was also found in the colon, despite lower abundance of these bacteria in this region (Sup. Fig. S2). Bacteria belonging to the Lachnospiraceae family showed similar trend as *Clostridium*, especially the genus *Dorea*, which was increased by HFS diet and restored by metformin in all intestinal segments (Sup. Fig S2). Furthermore, in the lumen of the 3 sections of the small intestine, bacteria belonging to the Peptostreptococcacae family also showed similar trend to *Clostridium*, with metformin reducing their abundance (Sup. Fig. S3).

In contrast, metformin treatment tended to increase *Adlercreutzia* abundance in the jejunum (Sup. Fig. S3). We also observed a positive effect of metformin on *Propionibacterium* (from the Actinobacteria phylum) in the duodenum (Sup. Fig. S3). Finally, there was a marked reduction of the Muribaculaceae family (S24-7) in response to HFS diet in the colon and metformin tended to further decrease the abundance of these bacteria (Sup. Fig. S3).

## Discussion

The mechanism of action of the antidiabetic drug metformin is still a matter of discussions although it is now widely accepted that the gut plays an important role^1^. Despite major advances, there are still uncertainties regarding the effects of metformin in the intestinal tract. In the present study, we aimed at gaining more insights into the mechanisms that occur in the different regions of the intestine in response to 8 days of metformin treatment in a mouse model of high-fat high-sucrose (HFS) diet. Adult male mice fed the HFS diet for 8 days displayed metabolic alterations, as evidenced by mild fasting hyperglycemia and reduced glucose tolerance. Metformin treatment was able to protect against these defects at the whole-body level.

### Effects of metformin on intestinal gene expression

Dissecting the molecular changes in the different regions of the gut, we found that metformin counteracted HFS diet-induced overexpression of a network of genes involved in the transport of glucose and fatty acids in the small intestine.

The effect of metformin on intestinal glucose transporters is not clear in the literature, although the general consensus is to consider that glucose transport from the intestinal lumen to the circulation is inhibited by metformin, together with an increased uptake and metabolism of glucose by enterocytes^1^. Mice fed the HFS diet were characterized by increased SGLT1 and GLUT5 mRNA levels in the duodenum, and we observed that metformin abolished these inductions, and also reduced GLUT5 expression in the ileum and the colon. These data pointed therefore towards a marked inhibition of sugar transporter expression, especially fructose transporter, after 8 days of metformin treatment, in agreement with a recent report showing reduced intestinal glucose absorption in mice in response to metformin^17^.

The impact of metformin on intestinal lipid transport is less documented. We observed a marked regulation of the expression of genes related to fatty acid transporters (CD36, FATP4, FABP2) and chylomicron synthesis (MTTP) in both ileum and jejunum, the preferential regions of lipid absorption. Metformin counteracted the overexpression induced by HFS diet or downregulated these genes. Inhibition of MTTP expression by metformin, associated with reduced chylomicron production, has been already observed in the *Psammomys obesus* sand rat model^18^. These data supported therefore an inhibition by metformin of HFS diet-induced lipid absorption, which may contribute, together with the reduction of sugar transporter expression and absorption^17^, to the improvement of metabolic homeostasis in treated animals.

Metformin treatment for 8 days also down-regulated the expression of genes coding for GIP and CCK in the duodenum, and for FGF15 in the ileum when compared to the HFS diet group. Fibroblast growth factor 15 (rodent ortholog of the human FGF19) has recently emerged as an important endocrine hormone involved in bile acids, lipid and glucose metabolism^19^. In agreement with our gene expression data, metformin was found to reduced serum FGF15 levels in a model of diabetic rats^20^, and decreased FGF19 levels was recently reported after 3 days of treatment in T2DM patients^21^. GLP-1 is regarded as one of the main mediators of metformin beneficial action on glucose homeostasis and many studies have reported increased glucagon gene expression in intestinal L-cells, as well as higher circulating levels of GLP-1 upon metformin treatment^1,9,22^. We did not observe here major modifications of glucagon gene expression in the different regions of the gut, suggesting that the regulation of GLP-1 expression is potentially not a short-term or a primary event in the mechanism of action of metformin in the HFS fed mouse model.

### Effects of metformin on microbiota

We evaluated the impact of metformin treatment on the luminal microbiota of each segment of the intestinal tract. The most striking and novel result was a strong induction by metformin of *Akkermansia muciniphila* abundance in all the sections of the gut. The effect of metformin on *A. muciniphila* has been already documented in faeces and stools both in rodent and in humans^10,12,14^. The novelty here was to show that this effect of metformin takes place in all the sections of the gut, including the upper small intestine. *A. muciniphila* classically uses mucins as energy source and leads to the degradation and renewal of the mucus layer, thus maintaining the intestinal barrier function^23^. However, we did not find modification of MUC2 expression in the different segments of the gut (Sup. Fig. S4), although this marker has been previously associated with mucus layer thickness in mice treated by metformin for 10 weeks^14^. This may suggest that other mechanisms could link *A. muciniphila* and metformin action. Interestingly, the beneficial metabolic effect of *A. muciniphila*, has been very recently associated with the production in the gut of mono-palmitoyl-glycerol species, members of the endocannabinoid family^24^. The regulation of these metabolites in the presence of metformin remains to be investigated.

In addition to *A. muciniphila*, we found that metformin reversed the induction by HFS diet of bacteria from the Lachnospiraceae family, including *Dorea,* and from the Clostridiaceae family, especially the genus *Clostridium*, in almost all the sections of the intestine, as well as from the Peptostreptococcacea family in the small intestine. Reduction in Lachnospiraceae abundance in response to metformin has been already observed in rodent studies^25^. Similarly, several members of the *Clostridium* genus have been found down-regulated in metformin-treated T2DM patients^13^. Members of these families are generally associated with compromised health conditions, like the Lachnospiraceaes that are increased in different diseases, including obesity and diabetes^26,27^. Our data may suggest a contribution of members of these families in HFS diet-induced metabolic alterations and in the beneficial effects of metformin.

Metformin treatment seemed also associated with an increase in the abundance of *Adlercreutzia* in the jejunum, with a tendency in the other parts of small intestine, and of *Propionibacterium* in the duodenum. Increased abundance of *Adlercreutzia* has been previously observed in some T2DM patients treated with metformin^28^. Interestingly, species of *Adlercreutzia* have been shown to metabolize isoflavonoids to equol that possesses strong antioxidant properties and can favourably affect various metabolic functions^29^. Regarding the *Propionibacterium*, several members possess beneficial properties, especially the ability to produce propionate and vitamin B12^30^. Increased fecal propionate concentration have been evidenced in T2DM patients treated with metformin^13^.

Beside *A. muciniphila*, additional specific bacteria were recently proposed as potential mediator of metformin action, but were not found in the present study, such as *Lactobacillus* members, that are increased after 1 day of metformin treatment in rat duodenum^31^, and *Bacteroides fragilis* which is decreased after 3 days of metformin in the stools of T2DM patients and that may regulate FXR signalling through the production of the bile acid GUDCA^21^.

### Effects of metformin on bile acid pool

The involvement of bile acids in the mechanism of action of metformin has recently emerged^11,21,22^. We found here that metformin did not significantly modify total bile acid levels in the caecum of the HFS diet fed mice, but altered their composition. The most striking effect of metformin was a marked decrease in the levels of the secondary bile acids DCA and LCA whereas UDCA and TUDCA were increased. DCA and LCA can be produced by gut bacterial 7α-dehydroxylation (7αDeOH) of the primary bile acids CA and CDCA. The predominant intestinal species exhibiting 7αDeOH activity belong to the genus *Clostridium*^32,33^. The fact that metformin significantly decreased their abundance in all the sections of the gut, may potentially support the observed reduction in DCA and LCA levels.

Ursodeoxycholic acid (UDCA) and its taurine conjugated TUDCA are of clinical interest due to their multiple beneficial effects on human health. CDCA can be converted to UDCA in the colon through epimerization by microbial 7α- and 7β-hydroxysteroid dehydrogenases. The bacteria bearing 7α/β-hydroxysteroid dehydrogenase activities are less characterized, but members of the genera *Clostridium* possess these activities^34^. UDCA is then transported from the intestine to the liver through the enterohepatic circulation where it is conjugated with taurine or glycine to produce TUDCA or GUDCA, which are transported back into the gut. We found increased levels of UDCA and TUDCA in the caecum of metformin treated mice, while GUDCA was not detectable (in agreement with the fact that there are very low levels of glycoconjugated bile acids in rodent). Our data in mice agreed therefore with a recent study showing that TUDCA and GUDCA are the most induced bile acids in the stool of T2DM patients treated for 3 days with metformin^21^. These authors also showed that UDCA, TUDCA and GUDCA are inhibitors of the nuclear receptor FXR^21^. Interestingly, DCA and LCA, which were increased in the present study, are also potential inhibitors of FXR^35^. Another study demonstrated that metformin could also directly inhibit FXR activity via AMPK activation^36^. Supporting an inhibition of FXR activity in metformin treated mice, we found a significant reduction in the expression level of FGF15, a major target gene of FXR in the ileum. Therefore, the contribution of FXR signalling in the action of metformin, directly or via bile acid modifications in the gut, is certainly an important mechanism to take into consideration.

Altogether the presented data demonstrate that metformin administrated during 8 days is able to counteract important perturbations induced by HFS diet in the different regions of the intestinal tract in mice, including altered expression of key genes of nutrient absorption and increased abundance of some gut bacteria, which have been associated with metabolic disturbances (f-Lachnospiraceae, f-Petostreptococcaceae, g-*Clostidium*). In addition, metformin promotes a strong increase in the abundance of *A. muciniphila* all along the gut, and beneficial modification of secondary bile acid profile in the caecum, with reduction in DCA and LCA levels and increased levels of UDCA and TUDCA, potentially leading to FRX inhibition. It will be now important to determine how these different events are interconnected and triggered by metformin. A limitation of the present work is the fact that metformin administration was initiated together with the HFS diet, therefore interfering with the adaptation of the mice to the diet. The results might thus by different in a therapeutic approach with metformin treatment provided in animals with already established metabolic alterations. Another limitation is the fact that the study did not include a control group to investigate the effects of metformin in mice fed the chow diet. Although such studies should be performed in the future, the present work clearly emphasises the role of all the regions of the intestine in the beneficial action of the antidiabetic drug metformin in prediabetic mice.

## Methods

### Animals, diet and metformin treatment

Twelve-week-old C57BL/6J/Ola/Hsd male mice (ENVIGO, Gannat, France) were maintained in a temperature-controlled (22 ± 2 °C) facility room with a 12 h light/dark cycle. Animals were allocated to 3 experimental groups (n = 5/6 animals per group) after one week of adaptation. The protocol was independently repeated 4 times. The control group (SD) was fed ad libitum with classical chow diet (R16, GENOBIOS, Laval, France), the HFS and HFS-MET groups were fed with a high-fat high-sugar diet (260HF, SAFE, Augy, France, composition shown in the Supplementary Table S1) for a period of 8 days while treated daily by intragastric gavage of metformin (300 mg/day/kg body weight) (HFS-MET) or water as control (HFS). Housing and experimentations were carried out in compliance with the ARRIVE guidelines and according to the French and European guidelines of laboratory animal care (European Communities Council Directive of 1986, 86/609/EEC). The protocol was approved by Rhône-Alpes Region institutional Ethics Committee for animal research (CECCAP) and registered under the reference CECCAP LS-2017-002.

Body weight was monitored at day 0 and day 8. For glucose tolerance test (ipGTT), mice were fasted for 6 h then received an intraperitoneal injection of glucose (2 g/kg body weight). Blood glucose was monitored at different time points during 90 min at the tip of the tail, using a glucometer (Accu-Check, ROCHE).

### Intestinal tissue sampling

Animals were killed by cervical dislocation after 6 h of fasting and 5 h after the last gavage with metformin. Different parts of the intestinal tract (duodenum, jejunum, ileum and the colon from medium part to anus) were sampled. The caecum was also removed and the intestinal segments were cleaned by flushing with ice-cold PBS and dipped in liquid nitrogen.

### Gene expression analyses in intestinal segments

Total RNA was extracted with TRI Reagent Solution (SIGMA). Target mRNA levels were measured by reverse transcription followed by real-time PCR using a Rotor-Gene (QIAGEN). A standard curve was systematically generated with different amounts of purified target cDNA, and each assay was performed in duplicate and normalized using TATA-binding protein mRNA level, as previously reported^37^. The list of the target genes with the PCR primers used for the qPCR assays is in Supplementary Table S2.

### Bile acid profiling in caecum

Bile acid molecular species concentrations were measured by HPLC coupled to tandem mass spectrometry (HPLC–MS/MS) as previously described^38^. Results were expressed in nmol/g of dried caecum.

### Microbiota analysis

Microbiota composition in the luminal content of the different intestinal sections was determined by 16S rRNA sequencing in 5 animals per group. At sacrifice, the different parts of the intestinal tract (duodenum, jejunum, ileum, colon) were carefully flushed 3 times with 5 ml of ice-cold PBS. The eluents were combined and centrifuged at 1800 rpm during 20 min at 4 °C. The supernatants were discarded and the pellets suspended in 300 µl of RNA later (SIGMA ALDRICH) and stored at − 80 °C until use. Total DNA was extracted with the QIAamp PowerFecal DNA Kit (QIAGEN). Library preparation and sequencing were outsourced to the Biomnigene company (https://www.biomnigene.fr/en/). A region of approximately 426 bp encompassing the V3 and V4 hypervariable regions of the 16S rDNA gene was targeted for sequencing. For the preparation of the libraries, “Illumina TruseqDNA Sample Preparation v2” was used. Sequencing was performed on Illumina Miseq2000. Quality check was performed by FASTQC and sequences were then trimmed, denoised and chimera filtered using the DADA2 plugin. Then, OTU annotation, taxonomic classification and analyses were performed using the Quantitative Insights Into Microbial Ecology (QIIME2 version 2019.4) platform^39^, using the fit-classifier-naive-bayes module with the default parameters and the Greengenes database (gg_13_8_otus.tar.gz classifier for 16S rRNA). On the 60 luminal samples prepared (4 intestinal sections of 5 animals per group), 10 samples were removed from the analysis because of too low number of reads after sequencing (< 80,000) and/or aberrant results showing the presence of only one bacteria family, not found in any other samples, reflecting thus possible amplification artifacts^40^. On the reminding 50 samples, a total of 6,504,328 valid reads were obtained.

### Statistical analyses

Data are presented as mean ± SEM. For the measured parameters, ordinary one-way ANOVA followed by Tukey’s multiple comparison test was performed using GRAPHPAD PRISM version 8.0. *p* < 0.05 was considered statistically significant.

## Supplementary Information

Supplementary Information.

## Data Availability

The datasets generated and analyzed during this study are available from the corresponding author on reasonable request.
