# Supplementary material for: Metformin treatment for 8 days impacts multiple intestinal parameters in high-fat high-sucrose fed mice
Source: Sci Rep. 2021 Aug 17;11:16684. doi: 10.1038/s41598-021-95117-0 (PMC8371110; doi:10.1038/s41598-021-95117-0)

SUPPLEMENTARY INFORMATION

Metformin treatment for 8 days impacts multiple intestinal parameters in high-fat high-sucrose fed mice

Amélie Bravard, Céline Gérard, Clémence Defois, Bérengère Benoit, Kassem Makki , Emmanuelle Meugnier, Dominique Rainteau, Jennifer Rieusset, Murielle Godet and Hubert Vidal

Supplementary Table 1 : composition of the 260HF diet

Custom Diets

SAFE CUSTOM DIETS

PRODUCT DATA SHEET
Release date: November 2020

SAFE® U8978 Version 19

Definition

260HF
Fats and sugars controlled custom diet for Rats & Mice

Ingredients

AMF butter, casein, maltodextrin, sucrose, pre-mixture of minerals PM AIN 93M_G 3,5%, soybean oil, pre-mixture of vitamins PV AIN 93M_G 1%, sodium bicarbonate, potassium citrate, DLmethionine, choline bitartrate, BHT Butylhydroxytoluene.

CENTESIMAL COMPOSITION

Animal Proteins	22.8 %	Others	0.002 %
Vitamins & Minerals	7.5 %		
Amino Acids	0.20 %		
Carbon Hydrates	33.65 %		
Oils & Fats	35.85 %		

NUTRITIONAL COMPOSITION

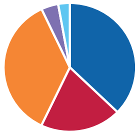

Nitrogen Free Extract	37.23 %
of which Starch	14.52 %
of which Sugars	20.25 %
Crude Protein	19.84 %
Crude Fat	35.92 %
Crude Ash	4.2 %
Crude Fiber	< 0.5 %
Moisture	2.8 %

ENERGY CONTENT

	MJ/kg	kcal/kg	%
ME Pig	22.7	5428.0	
ME Atwater	23.1	5515.9	
Energy from proteins	3.3	793.7	14.4
Energy from lipids	13.5	3233.0	58.6
Energy from NFE	6.2	1489.2	27.0

More information on energy calculation: www.safe-lab.com

For the welfare of animals SAFE® bedding and environmental enrichment such as SAFE® block gnawing logs and SAFE® nesting materials should be available in the cage.

Theorical Calculated Values

TOTAL PER KG

AMINO ACIDS

Arginine	7 850 mg	Methionine	8 221 mg
Cystine	818 mg	Tryptophan	2 429 mg
Lysine	17 084 mg	Glycine	3 943 mg

FATTY ACIDS

Palmitic acid	110 471 mg	EPA	90 mg
Stearic acid	37 134 mg	DPA	147 mg
Palmitoleic acid	4 084 mg	Sum SFA	233 125 mg
Oleic acid	63 883 mg	Sum UFA	93 825 mg
LA	16 894 mg	Sum MUFA	70 995 mg
ALA	3 019 mg	Sum PUFA	22 831 mg
Sum n-3	3 256 mg	Cholesterol	870 mg
Sum n-6	18 718 mg		

MINERALS

	END PRODUCT
Calcium	6 461 mg
Phosphorus	3 373 mg
Sodium	4 243 mg
Potassium	6 379 mg
Magnesium	841 mg
Manganese	15 mg
Iron	68 mg
Copper	8.1 mg
Zinc	55 mg
Chlorine	1 932 mg

VITAMINS

	END PRODUCT
Vitamin A	5 649 IU
Vitamin D3	1 625 IU
Vitamin E	120 IU
Vitamin K3	8.0 mg
Vitamin B1	7.8 mg
Vitamin B2	7.6 mg
Vitamin B3	45 mg
Vitamin B5	21 mg
Vitamin B6	9.1 mg
Vitamin B9	2.6 mg
Vitamin B12	0.033 mg
Biotin	0.26 mg
Choline	825 mg
Vitamin C	< 10 mg

SUGARS

Glucose	< 0.5 %	Lactose	< 0.5 %
Sucrose	18 %		

The values of the end products are given as indication only and have no contractual value. They are theorical calculated values of the diet formula without considering values from customer's compounds. Depending on production conditions, storage and analytical methods variations may occur. An analysis is performed on request.

Produced in France

SAFE COMPLETE CARE COMPETENCE
WORLDWIDE HEADQUARTERS
73494 Rosenberg (Germany)
service@safe-lab.com
DIETS PRODUCTION SITE
89290 Augy (France)
info@safe-lab.com
www.safe-lab.com

Supplementary Table S2 : List of target genes and qPCR primers

Name	Symbol	Forward primer sequence	Reverse primer sequence
**Nutrient transporters**			
Sugars			
SGLT1 (sodium-glucose transporter 1)	Slc5a1	**GCTCCTTGACCTCCATCTTC**	**CAGGCAATGCTGATGCCAAT**
GLUT5 (glucose/fructose transporter 5)	Slc2a5	**ATCACTGTCGGCATCCTTGT**	**TCTTCTGGATCAGCAGGTAG**
Fatty acids			
FAT/CD36 (fatty acid transporter)	Cd36	**GTCCTGGCTGTGTTTGGAGG**	**AAGATCCAAAACTGTCTGTA**
FATP2 (fatty acid transport protein 2)	Slc27a2	**ACCTCTCGGACAGCAATCAG**	**CTTTCCCTACAGTCTAGCAG**
FABP4 (fatty acid binding protein 4)	Fabp4	**GACACCTCAAAGGCACGAGC**	**GAGGGTCCAGATGCTCTGTG**
Cholesterol			
NCPC1L1 (NPC1 like intracellular cholesterol transporter 1)	Npc1l1	**GCTAGCAGCCAACATCACAG**	**CAGTAGGAGGTAGCAGACCA**
Bile acids			
ASBT (apical sodium/bile acid cotransporter)	Slc10a2	**ACCTCAGTGTTAGCATGACC**	**TTCAGGACAGGGGTAACCAC**
OSTα (bile acide transporter)	Slc51a	**AGAACACCCTTTGCCCCATC**	**CAAAGCCTTCCACCATGACC**
**Chylomicron synthesis**			
MTTP (microsomal triglyceride transfer protein)	Mttp	**GGAGAAGTAACCTGAACATC**	**ACAGGTCTGAGCTGAACATC**
**Gut hormones**			
Gastric inhibitory polypeptide	Gip	**GTGGCTTTGAAGACCTGCTC**	**AAGTCCCCTCTGCGTACCTT**
Cholecystokinin	Cck	**ACTGCTAGCGCGATACATCC**	**ATCCATCCAGCCCATGTAGT**
Neuropeptide Y	Npy	**CCTGAGACACTGATTTCAGA**	**GTGGTGGCATGCATTGGTG**
Glucagon/Glucagon like peptide 1 (GLP1)	Gcg	**GCCGTGCCCAAGATTTTGTG**	**CCTTTCACCAGCCAAGCAATG**
Fibroblast growth factor 15	Fgf15	**GAGGAGGACCAAAACGAACG**	**GAAGGTACAGTCTTCCTCCG**
**Inflammation**			
Interleukin 1 beta	IL1β	**ACTGTTCCTGAACTCAACTG**	**CTTGTTGATGTGCTGCTGCG**
Tumor necrosis factor alpha	TNFα	**CCAGACCCTCACACTCAGATC**	**CACTTGGTGGTTTGCTACGAC**

LEGENDS OF SUPPLEMENTARY FIGURES

Supplementary Figure S1: Number of Operational Taxonomic Units (OTUs) measured in the different sections of the intestinal tract. The number of OTUs gave an estimate of the bacterial richness, which appeared globally not affected by metformin in the 4 investigated regions. * $p < 0.05$.

Supplementary Figure S2: Effects of HFS diet and of metformin on the abundance of specific bacteria in the luminal effluent of the intestine (part I).
Supplemental Figure S2A: data expressed as number of counts in the 16S rRNA sequencing data results.
Supplemental Figure S2B: data expressed as relative abundance (percentage).
* $p < 0.05$, ** $p < 0.01$, *** $p < 0.001$, determined by ANOVA followed by Tukey's multiple comparison test.

Supplementary Figure S3: Effects of HFS diet and of metformin on the abundance of specific bacteria in the luminal effluent of the intestine (part II).
Supplemental Figure S3A: data expressed as number of counts in the 16S rRNA sequencing data results.
Supplemental Figure S3B: data expressed as relative abundance (percentage).
* $p < 0.05$, ** $p < 0.01$, *** $p < 0.001$, determined by ANOVA followed by Tukey's multiple comparison test.

Supplementary Figure S4 : Expression of Mucin 2 (MUC2) mRNA in the different regions of the intestine. The mRNA levels of MUC2 were determined by RT-qPCR in (**a**) the duodenum, (**b**) jejunum, (**c**) ileum and (**d**) colon of 10 to 12 mice per group. (SD : standard diet; HFS : high fat high sucrose diet; HFS-MET : HFS diet with metformin supplementation). *** $p < 0.001$, determined by ANOVA followed by Tukey's multiple comparison test.

Supplementary Figure S1

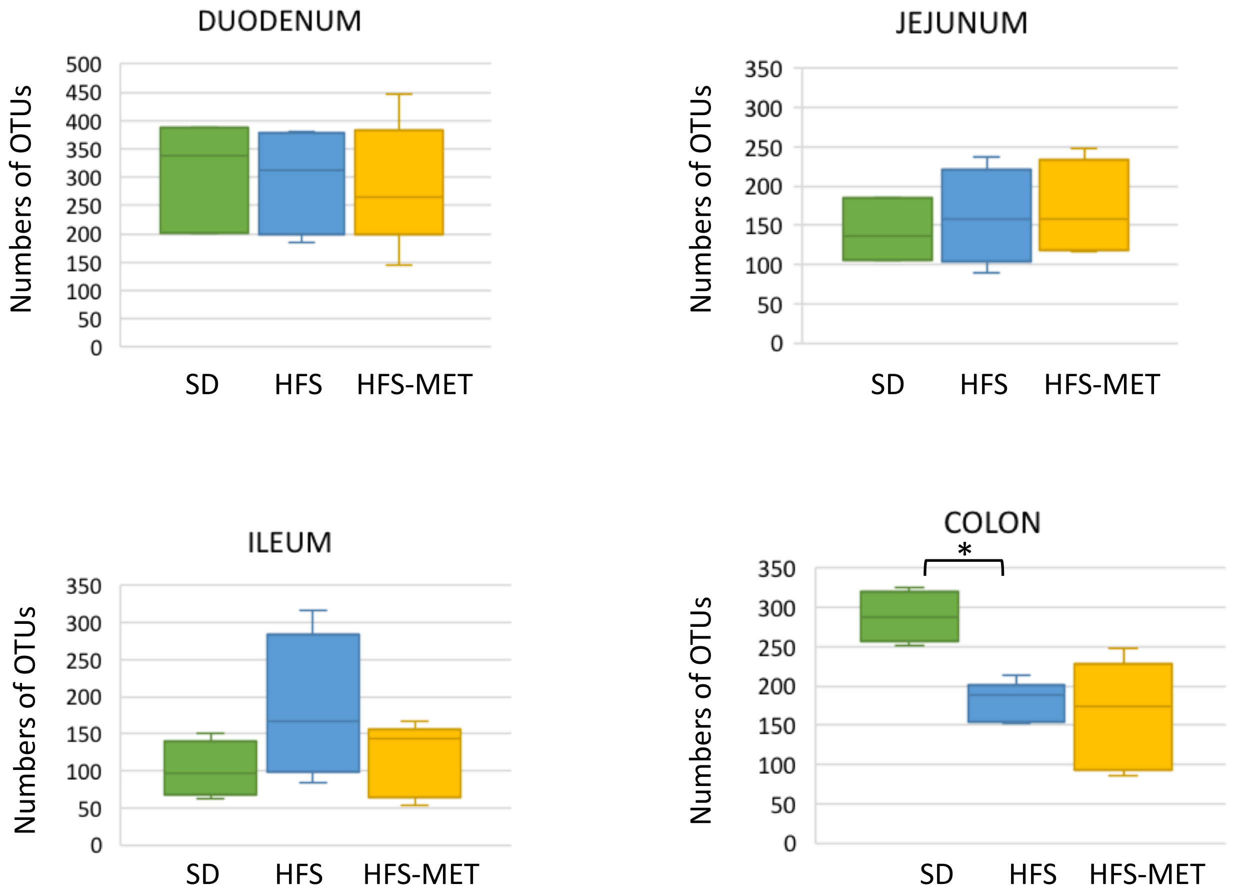

Supplementary Figure S2A (with data presented as number of counts)

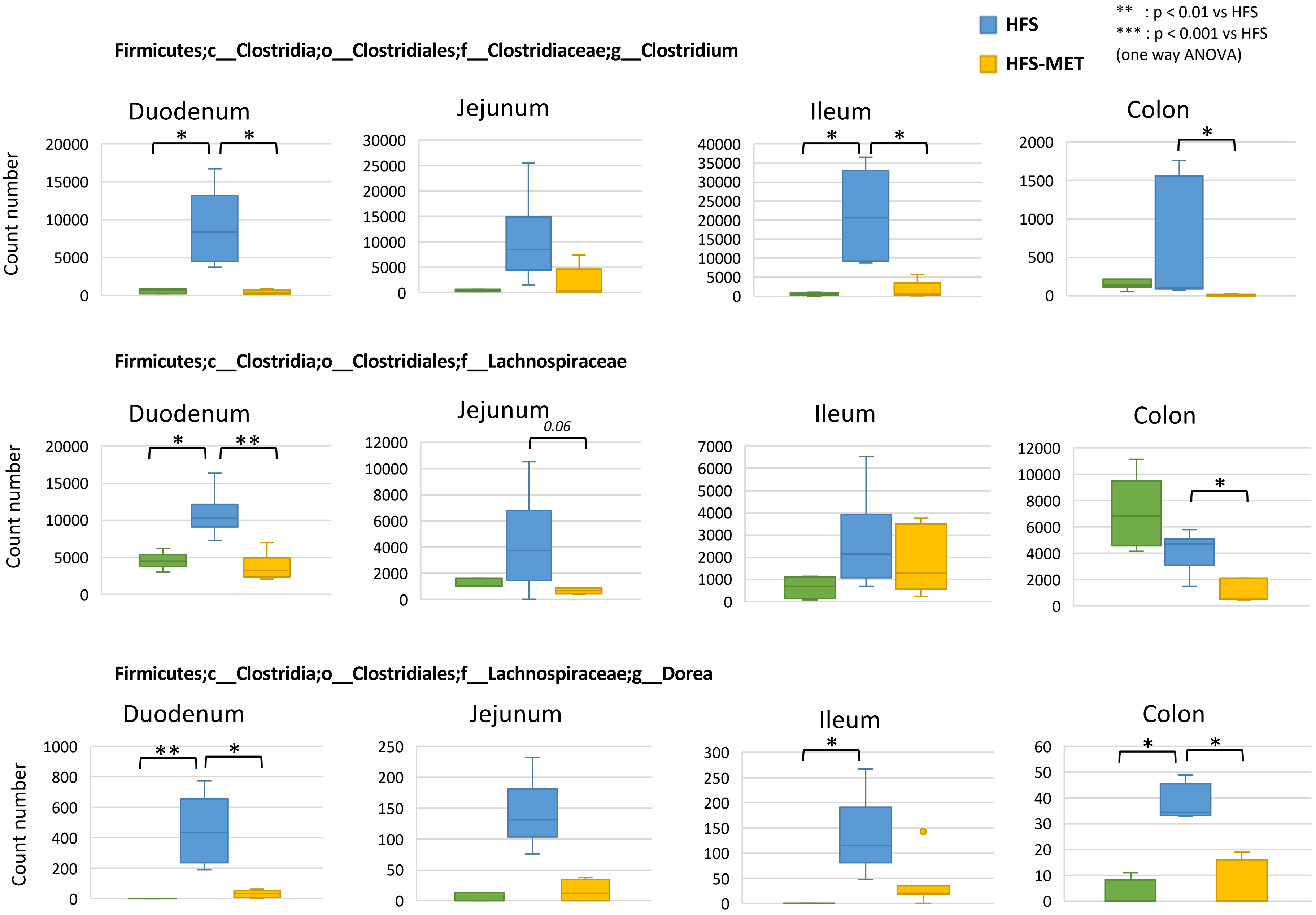

Supplementary Figure S2B (data presented as relative abundance (%))

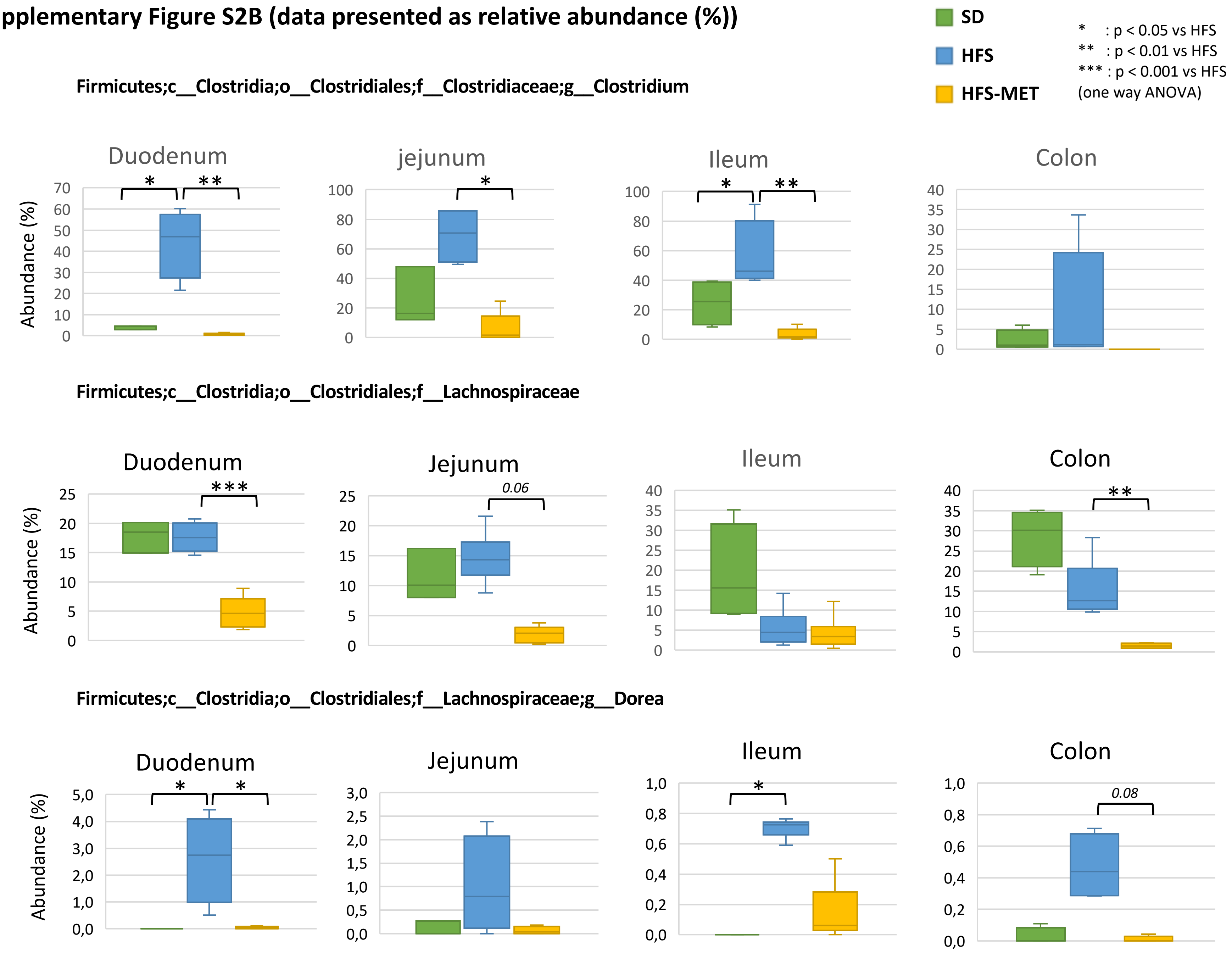

Supplementary Figure S3A (data presented as number of counts)

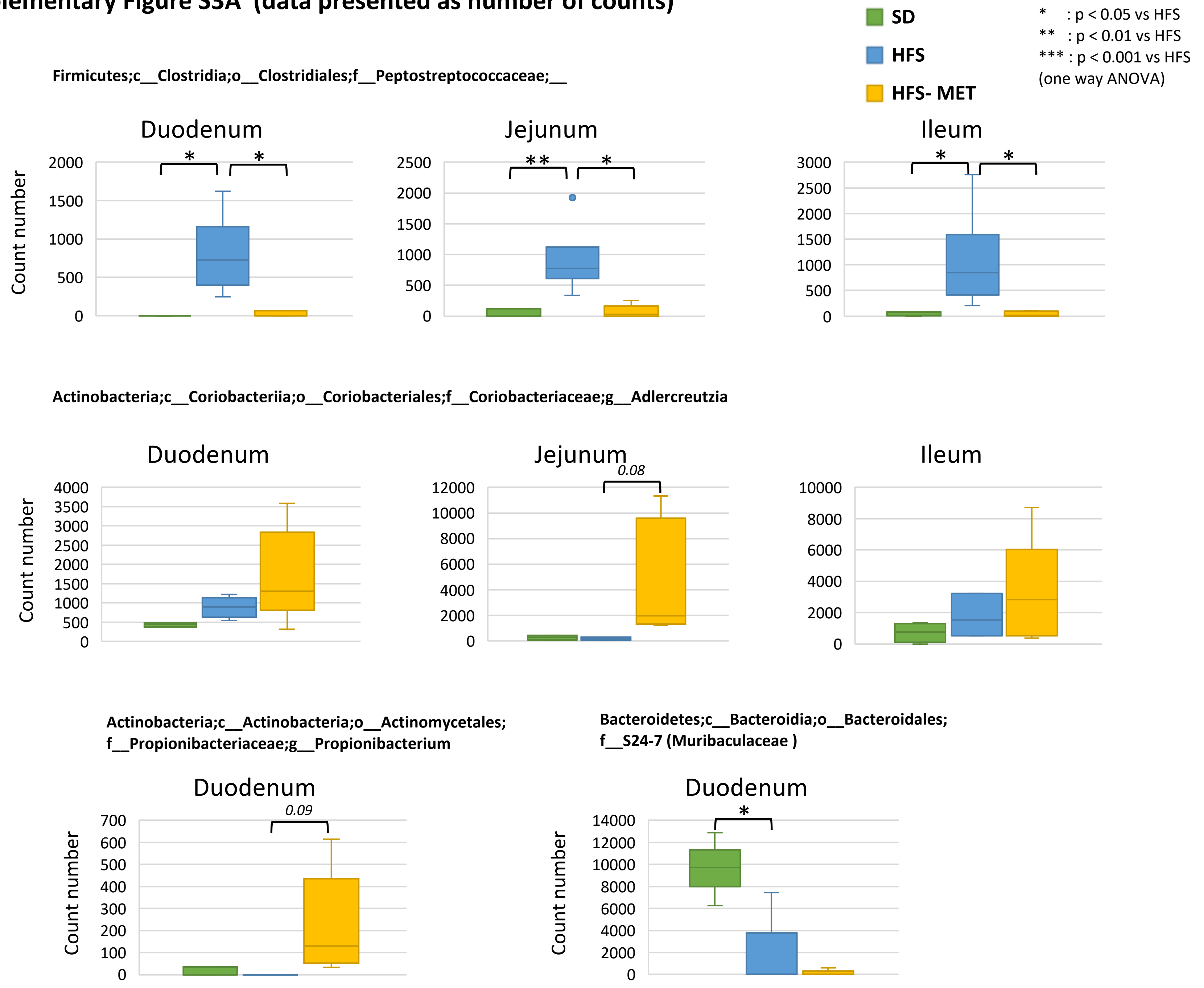

Supplementary Figure S3B (data presented as relative abundance (%))

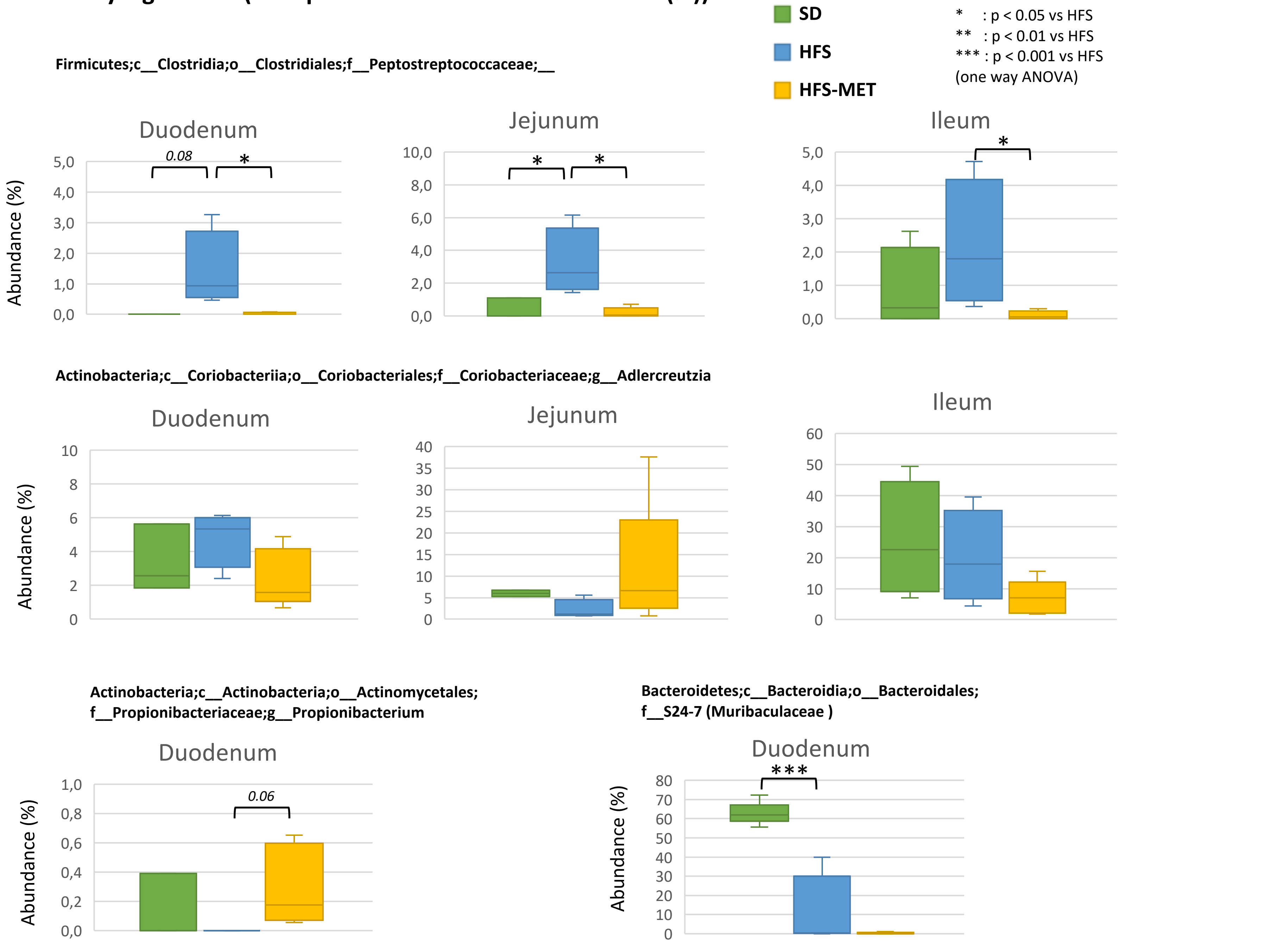

Supplementary Figure S4

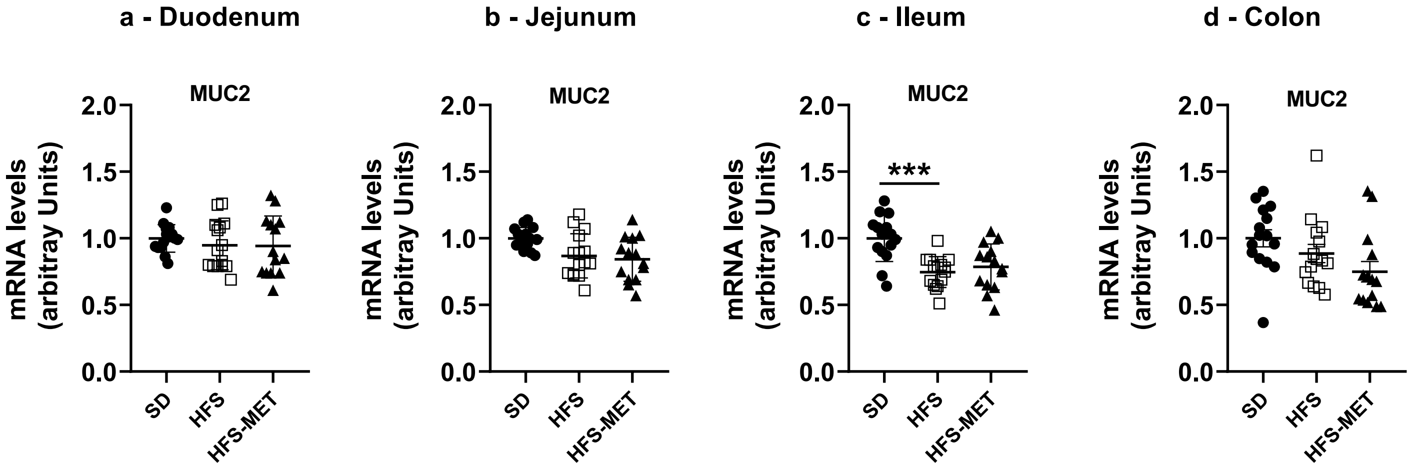

Supplement: Supplementary file 1 — Supplementary Information. [file 41598_2021_95117_MOESM1_ESM.pdf]
